# The antibacterial efficacy and effect of tungsten nanoparticles (WO_3_) on the expression of tetracycline and erythromycin-resistance genes in Streptococcus agalactiae isolated from pregnant women

**DOI:** 10.3205/dgkh000569

**Published:** 2025-07-11

**Authors:** Ava Salehi, Mohammad Karim Rahimi, Fatemeh Bagheri

**Affiliations:** 1 Department of Microbiology, Medical Faculty, Islamic Azad Medical University, Tehran, Iran; 2 Department of Basic Sciences, Faculty of Pharmacy and Pharmaceutical sciences, Tehran Medical sciences, Islamic Azad University, Tehran, Iran

**Keywords:** tungsten oxide nanoparticle, tetracycline resistance, erythromycin resistance, S. agalactiae

## Abstract

**Background and objectives:**

Colonization of pregnant women by *Streptococcus (S.) agalactiae* can lead to intrauterine infections after childbirth and potentially life-threatening infections in newborns. The current effectiveness of available antimicrobials is decreasing, posing a serious threat. Hence, there is an urgent requirement to develop novel categories of antimicrobial agents that can efficiently and swiftly eradicate these infections. To developed new strategies in the management and reduction of infections arising from *S. agalactiae*, our objective was to evaluate the antibacterial efficacy of tungsten nanoparticles (WO_3_) on the expression of tetracycline and erythromycin-resistance genes in *S. agalactiae* isolated from pregnant women.

**Materials and methods:**

A total of 46 Group-B streptococcus (GBS) isolates from rectovaginal swabs, blood, and urine cultures were obtained from pregnant women (13–35 weeks gestation) attending Central and Gynecological Hospitals in Tehran, Iran. The identification of GBS isolates was conducted using a variety of routine bacteriological techniques and targeted assays for the molecular characterization of the GBS isolates. The antimicrobial susceptibility test was carried out according to the Kirby-Bauer method. PCR was employed to screen for the presence of tetracycline and erythromycin resistance-associated genes. Tungsten oxide (WO_3_) nanomaterials were successfully synthesized and characterized using FE-SEM (field emission scanning electron microscopy), and DLS (dynamic light scattering) techniques. The microdilution assay was used to assess the antimicrobial efficacy of WO_3_ nanostructures. Furthermore, real-time PCR was employed to investigate the effectiveness of WO_3_ nanostructures in the regulation of the expression of the *tetM* and *ermB* resistance genes.

**Results:**

The findings of the antibiotic susceptibility assays demonstrated a considerable proportion of *S. agalactiae* strains with high resistance to tetracycline (87%), erythromycin (71.4%), and clindamycin (63%). Conversely, the resistance rates for chloramphenicol and levofloxacin were 8.7% and 6.5%, respectively. The results of antibiotic susceptibility assays revealed high-resistance *S. agalactiae* strains to tetracycline (87%), erythromycin (71.4%), and clindamycin (63%), while resistance rates chloramphenicol, levofloxacin, penicillin and ampicillin were 33.3%, 14.8%, 11.1%, and 7.4%, respectively. In addition to the mentioned antibiotics, it is worth noting that all strains exhibited sensitivity to other antibiotics such as ceftriaxone, linezolid, and vancomycin. Of the 24 (88.8%) erythromycin-resistant/intermediate isolates, the *ermB* gene was found in 16 (66.6%), and the *mefA* gene in 1 (4.2%) isolates. Furthermore, the *tetM* and *tetO* genes were recovered by 83.3% and 4.2% of the tetracycline-resistant isolates, respectively. By utilizing FE-SEM and DLS techniques, it was estimated that the average size of the WO_3_ nanomaterials were 100 nm and 51.2 nm, respectively. WO_3_ displayed varying effectiveness against 27 *S. agalactiae* strains, with minimal inhibitory concentration (MIC) ranging from 500 to 1,000 µg/mL. In addition, the application of nanostructures induced a considerable down-regulation of the antibiotic resistance genes *(tetM*, *ermB)* relative to the untreated isolate.

**Conclusion:**

The findings indicate that tungsten trioxide nanoparticles hold the potential to serve as a promising pathway for the development of new antibacterial substances, with the specific aim of addressing the problem of antibiotic resistance in infections caused by *S. agalactiae*.

## Zusammenfassung

**Zielsetzung:**

Die Kolonisierung schwangerer Frauen durch *Streptococcus (S.) agalactiae* kann nach der Geburt zu intrauterinen Infektionen und potenziell lebensbedrohlichen Infektionen bei Neugeborenen führen. Die derzeitige Wirksamkeit verfügbarer Antimikrobiotika nimmt ab und stellt eine ernsthafte Bedrohung dar. Daher besteht dringender Bedarf an der Entwicklung neuartiger Klassen antimikrobieller Wirkstoffe, die diese Infektionen effizient und schnell bekämpfen können. Zur Entwicklung neuer Strategien zur Bekämpfung von Infektionen durch *S. agalactiae* sollte die antibakterielle Wirksamkeit von Wolfram-Nanopartikeln (WO_3_) auf die Expression von Tetracyclin- und Erythromycin-Resistenzgenen bei *S. agalactiae*, die von schwangeren Frauen isoliert wurden, bewertet werden.

**Material und Methode:**

Es wurden 46 Gruppe-B-Streptokokken (GBS)-Isolate aus Rektovaginalabstrichen, Blut- und Urinkulturen von schwangeren Frauen (13.–35. Schwangerschaftswoche) in den Zentralen und Gynäkologischen Krankenhäusern in Teheran, Iran, untersucht. Die Identifizierung der GBS-Isolate wurde mithilfe verschiedener routinemäßiger bakteriologischer Techniken und gezielter Assays zur molekularen Charakterisierung der GBS-Isolate durchgeführt. Das Antibiogramm wurde gemäß der Kirby-Bauer-Methode bestimmt. Mittels PCR wurde das Vorhandensein von Tetracyclin- und Erythromycin-Resistenz-assoziierten Genen gescreent. WO_3_ Nanomaterialien wurden synthetisiert und mithilfe von FE-SEM (Rasterelektronenmikroskop) und DLS (dynamische Lichtstreuung) charakterisiert. Die antimikrobielle Wirksamkeit der WO_3_-Nanostrukturen wurde im Mikrodilutionstest bestimmt. Darüber hinaus wurde die Echtzeit-PCR eingesetzt, um die Wirksamkeit von WO_3_-Nanostrukturen bei der Regulation der Expression der Tetracyclin- und Erythromycin-Resistenzgene zu untersuchen.

**Ergebnisse:**

Die Ergebnisse des Antibiogramms zeigten, dass ein beträchtlicher Anteil der *S. agalactiae*-Stämme eine Resistenz gegen Tetracyclin (87%), Erythromycin (71,4%) und Clindamycin (63%) aufwies. Die Resistenzraten für Chloramphenicol und Levofloxacin waren hingegen nur 8,7% bzw. 6,5%. Die Ergebnisse der Antibiotikaempfindlichkeitstests zeigten hohe Resistenz gegen Tetracyclin (89%), Erythromycin (70,3%) und Clindamycin (63%), während die Resistenzraten für Chloramphenicol, Levofloxacin, Penicillin und Ampicillin bei 33,3%, 14,8%, 11,1% bzw. 7,4% lagen. Zusätzlich zu den genannten Antibiotika sei darauf hingewiesen, dass alle Stämme empfindlich auf andere verwendete Antibiotika wie Ceftriaxon, Linezolid und Vancomycin reagierten. Von den 24 (88,8%) Erythromycin-resistenten/intermediären Isolaten wurden das *ermB*-Gen in 16 (66,6%) Isolaten und das *mefA*-Gen in eine (4,2%) Isolat gefunden. Darüber hinaus wurden das *tetM*- und *tetO*-Gen in 83,3% bzw. 4,2% der Tetracyclin-resistenten Isolaten nachgewiesen. Mithilfe von FE-SEM- und DLS-Techniken konnte geschätzt werden, dass die durchschnittliche Größe der WO_3_-Nanomaterialien bei 100 nm bzw. 51,2 nm lag. WO_3_ zeigte unterschiedliche Wirksamkeit gegen 27 *S. agalactiae*-Stämme, wobei die minimale Hemmkonzentration (MIC) im Bereich von 500 bis 1.000 µg/mL lag. Darüber hinaus führte die Anwendung der Nanostrukturen zu erheblicher Herunterregulierung der Antibiotikaresistenzgene (*tetM*, *ermB*) im Vergleich zu den unbehandelten Isolaten.

**Fazit:**

Die Ergebnisse zeigen, dass Nanopartikel aus Wolframtrioxid (WO_3_-NPs) das Potenzial haben, eine vielversprechende Möglichkeit für die Entwicklung neuer antibakterieller Substanzen darzustellen, die speziell darauf abzielen, das Problem der Antibiotikaresistenz bei Infektionen durch *S. agalactiae* anzugehen.

## Introduction

*Streptococcus (S.) agalactiae*, commonly referred to as Group B Streptococcus (GBS), is a Gram-positive coccus found in the intestinal and genital tracts of most normal individuals [1]. This micro-organism was not recognized until the late 1960s, but was later described as the causative agent of infections in infants and their mothers in the United States and Europe [1]. GBS may lead to various problems, including skin and soft tissue infections, sepsis, meningitis, pneumonia and endocarditis. These infections tend to affect young children, pregnant women and people with co-existing disorders such as diabetes, nerve damage, cancer and cirrhosis of the liver. The genital tract provides a supportive environment for *S. agalactiae* growth and reproduction. However, 70–80% of infected mothers may have vertical transmission of the bacterium to their infants. GBS is associated with both early onset disease (EOD) and late onset disease (LOD) in babies [2]. The most notable factors that increase the risk of infection with GBS are: initiating labor prior to the 37^th^ week of pregnancy, experiencing premature rupture of placenta during the first 18 hours prior to gestation, presenting a fever exceeding 38°C during pregnancy, having an older infant affected by invasive illness complicated by GBS, and having a current delivery with a prior experience of GBS-related urinary tract infection [2]. Despite the proven efficacy of intrapartum antibiotic prophylaxis (IAP), GBS currently accounts for 150,000 stillbirths and infant mortalities globally, as stated by the World Health Organization (WHO) [3]. The application of IAP is based on pre-determined guidelines, which may be derived from either risk factors or screening protocols. Consequently, it is of utmost significance to monitor rates of antibiotic resistance. Penicillin serves as the primary antibiotic for IAP. In severe penicillin hypersensitivity, the utilization of second-line antibiotics, such as macrolides (erythromycin) and lincosamides (clindamycin), are recommended. Nevertheless, the employment of these antibiotics is constrained due to the escalating resistance against both. Although the effectiveness of penicillin against GBS is still recognized, a growing body of evidence indicates the presence of isolates that display diminished susceptibility to this antibiotic. This occurrence has raised significant concerns, particularly when resistance to second-line antibiotics becomes an established characteristic of GBS [4], [5]. Furthermore, the dissemination of resistant pathogens among both human and animal populations, as well as the potential contamination of the environment through the utilization of manure as fertilizer, contributes to the heightened global risk from a One Health perspective [6]. Erythromycin resistance occurs due to the methylation of ribosomes by methyltransferases encoded by erm genes *(ermB*, *ermA/TR*). Another reason for resistance is the heightened expression of efflux pumps, which expel the drug without altering it. Macrolide efflux pumps (Mef) are encoded by *mefA/E* genes and contribute to macrolide resistance in group B *streptococci* [7], [8]. Tetracycline resistance in GBS primarily arises from the presence of specific resistance protein pumps (RPPs). The key RPPs associated with tetracycline resistance in GBS are *TetK*, *TetL* *TetM*, and *TetO*, which actively expel tetracycline from the bacterial cell, conferring tetracycline resistance in GBS [9]. 

The increasing occurrence of antibiotic resistance is a matter of great concern. Consequently, scientists are investigating and formulating novel antimicrobial agents to counteract infections brought about by pathogens that are resistant to multiple drugs [10]. The healthcare industry is currently investigating methods that are non-toxic and non-invasive in order to manage and prevent infection [11]. Non-toxic metal biomaterials may potentially eliminate the need for conventional antimicrobial agents [11]. The formulation and administration of non-toxic, biocompatible metal materials that possess antimicrobial attributes, including materials that are utilized in the production of pharmaceuticals and medical devices, could potentially eliminate the necessity for traditional antimicrobial agents [12], [13]. Inorganic antimicrobial compounds are commonly preferred due to their tendency to possess non-specific toxicity mechanisms, resilience in severe conditions, and affordability [14], [15]. Specifically, tungsten-oxide (also known as wolfram oxide, WO_x_) nanomaterials display stable physiochemical properties and have been shown to exhibit reduced toxicity towards mammalian cells [14]. The effective photocatalytic response of nano-crystalline tungsten oxide is observed even when it is excited by photons from visible light [16]. The electron-hole pair is generated by the photon, which leads to the production of free radicals that undergo subsequent oxidation and reduction reactions. Consequently, nanometric WO_3_ particles with fine crystallinity can be utilized for cost-effective wastewater treatment, degradation of harmful dyes, and other organic compounds using a straightforward visible light source or sunlight. Furthermore, nanometric tungsten oxides are acknowledged for their potent antibacterial effects [14], [17]. Extremely small tungsten oxide nanoparticles can exterminate bacteria either by directly penetrating the cell membrane or by releasing metal ions. The antibacterial effect may be strengthened under light exposure, as photocatalysis generates reactive oxygen species (ROS), which can hinder bacterial growth by inducing oxidative stress [14], [15]. Hence, the part played by nanoparticles in overcoming antibiotic resistance is a matter of great importance. In the present study, we assessed the antibacterial characteristics of tungsten nanoparticles (WO_3_) and their effects on the expression of tetracycline and erythromycin-resistance genes in *S. agalactiae* isolated from pregnant women.

## Materials and methods

### GBS strain collection and identification

Between September 2021 and April 2022, a total of 146 vaginal-swab specimens were collected for screening GBS isolates from different pregnant women aged between 20 and 40 years at 35–37 weeks of pregnancy at different teaching hospitals and private centers in Tehran, Iran. The identification of bacterial species was accomplished using established methods. Upon obtaining informed consent, one vaginal sample was procured from each patient, which was then placed in an Amies transport tray and stored at 4°C. The samples were subsequently placed in a selective enrichment medium (Todd-Hewitt broth, Oxoid, supplemented with nalidixic acid 15 µg/ml and colistin 10 µg/mL). Following an incubation period of 18–24 hours at 37°C, the broth cultures were subjected to further cultivation on 5% blood agar and incubated for an additional 18–24 hours at 37°C. The identification of colonies was carried out through the utilization of Gram staining, haemolysis, catalase, and CAMP [18].

### Antimicrobial susceptibility testing

The modified Kirby-Bauer disc diffusion technique was applied to screen for antibacterial susceptibility in accordance with the CLSI 2020 guidelines [19]. The bacterial suspension was placed on Mueller-Hinton agar which was supplemented with 5% sheep blood. The antibiotics that were subjected to testing (Oxoid Ltd., Basingstoke Hampshire, England) comprised penicillin (10 U), ampicillin (10 µg), erythromycin (15 µg), clindamycin (2 µg), chloramphenicol (30 µg), levofloxacin (5 µg), ceftriaxone (30 µg), tetracycline (30 µg), Linezolid (30 µg), and vancomycin (30 µg). The Clinical and Laboratory Standards Institute (CLSI) guidelines were followed for both the preparation of the inoculum and the interpretation of the susceptibility test results. *S. pneumoniae* ATCC 49619 and *Staphylococcus aureus* ATCC 25923 were used as quality control strains.

### DNA extraction

The method utilized to extract DNA from all *S. agalactiae* isolates involved a commercial DNA extraction kit (Cinna Gen. Co) in accordance with the guidelines provided by the manufacturer. Subsequently, the DNA samples were subjected to agarose gel electrophoresis with a concentration of 1, followed by staining with ethidium bromide. The samples were observed using a UV transilluminator. The DNA assessed by applying Nano Drop at 260/280 nm at 0.5°C. The DNA was then preserved at –20°C for subsequent experiments.

### Determination of resistance genes

Tetracycline and erythromycin resistance genes *tetM*, *tetO*, *ermB*, and *mefA*, as well as the identification of potential *S. agalactiae* isolates, were detected using a PCR assay that specifically focused on the housekeeping gene referred to as *16 s rRNA*. The primers employed in this process are listed in Table 1 (Tab. 1). The reaction mixture was prepared as follows: 10 µL of 2X Taq Premix-Master mix (Yekta Tajhiz Co, Iran), 6 µL of nuclease-free water, 1 µL of each forward primer, 1 µL of each reverse primer, and 2 µL of DNA template were combined, resulting in a final volume of 20 µL. The PCR conditions for both reactions were based on the method suggested in Mudzana et al. [20]: 94°C for 1 min (initial denaturation), 35 cycles [94°C for 1 min (denaturation) 55°C for 1 min (annealing) and 72°C for 1 min (extension)], and a final extension step at 94°C for 1 min. The resulting amplicons were electrophoresed on a 1.5% agarose gel in 1X TAE buffer at 100 V for 60 min. The gel was stained with ethidium bromide (5 µg/100 mL) and visualized using a UV transilluminator (Cambridge, UK).

### Preparation of tungsten oxide (WO_3_) nanoparticles

The WO_3_ nanoparticles were fabricated through a chemical precipitation method. To synthesize these nanostructures, sodium tungstate was dispersed in 25 mL of distilled water. Subsequently, 2.5 mL of 2 mol HCl was gradually dropped into the solution containing sodium tungstate while maintaining continuous stirring. By maintaining controlled and continual stirring at 500 rpm and a temperature of 25°C for 3 hours, the precursor underwent a transformation, becoming dense and acquiring a yellow hue. The resulting precipitate was then subjected to filtration and rinsed repeatedly with deionized water (DI). Following this, it was dried for 2 hours at 120°C in an oven. Ultimately, the WO_3_ nanoparticles were calcined at a temperature of 300°C for 3 hours in a programmable furnace, and the dried precipitate was ground into a fine powder [21].

### Characterization of WO_3_ nanostructures

A field emission scanning electron microscope (FE-SEM) was utilized to assess the surface morphological features and dimensions of tungsten trioxide nanoparticles (WO_3_ NPs). Prior to analysis, a conductive gold layer was applied to the samples to prevent problems related to charging during observation. The hydrodynamic size and zeta potential analyses were executed employing the dynamic light scattering (DLS) method using a Malvern- DTS Ver 4.20. 

### Antibacterial efficacy

To determine the minimum inhibitory concentration (MIC) of tungsten nanoparticles against strains of *S. agalactiae*, the microbroth dilution method was utilized, following the guidelines set by the Clinical and Laboratory Standards Institute (CLSI). Initially, colonies of *S. agalactiae* were cultured in Mueller Hinton Broth (MHB) and incubated at 37°C for 24 hours under continuous stirring at a speed of 180 rpm. Subsequently, the number of bacterial cells was adjusted using a spectrophotometer at a wavelength of 625 nm to achieve a standardized turbidity of 0.5 McFarland. In this study, the accuracy of the bacterial count was improved by setting the optical density (OD) of the bacterial suspension to 0.09. The bacterial suspension was then diluted in MHB to obtain a colony count of 10^6^ CFU/mL. Furthermore, the nanoparticles underwent serial dilution in MHB using a volume of 100 µL in a 96-well microplate (25, 50, 100, 500, 1,000 µg/mL). Ultimately, 100 µL of the bacterial suspension, equivalent to 10^5^ CFU, was inoculated with each of the diluted antibiotics. The microplates were subsequently incubated at 37°C for 18 to 24 hours. As per the definition established by the CLSI [19], the MIC represents the lowest concentration of antimicrobial that completely inhibits the growth of bacteria. The MIC assays were conducted at least three times for all 27 strains. In order to assess the antimicrobial efficacy of the biomaterials synthesized prior to experimentation, a colony count assay was conducted on 5 strains of MDR *S. agalactiae*. The bacterial cultures were initially prepared in phosphate buffer saline (PBS) at a density of 0.5 McFarland, which is equal to 10^8^ CFU/mL, and then further diluted in PBS to a concentration of 10^3^ CFU/mL. The antibacterial properties were evaluated by placing the powder samples in molten Mueller-Hinton agar at various concentrations under agitation. Upon reaching 55°C, the homogeneous solutions were poured into Petri dishes, solidified, and 100 µL of bacterial solution was spread on the agar plates containing different quantities of WO_3_, with unloaded samples serving as the control [14], [22]. Following incubation at 37°C for 24–48 hours to facilitate bacterial proliferation, the colonies were quantified digitally with the aid of colony counting software.

### RNA extraction and quantitative real-time PCR

Real-time PCR was used to assess the effect of sub-inhibitory concentrations of tungsten nanoparticles on the expression of *tetM* and *ermB* genes in 5 MDR strains of *S. agalactiae*. Treated and untreated cultures of *S. agalactiae* were obtained by centrifuging at 6,000 x g for 15 minutes at 4°C. The collected bacterial pellets were then resuspended in 100 µL of Tris-EDTA buffer containing lysozyme. The pellets were kept at 25°C for 5 minutes before lysis using RNA lysis buffer. Total RNA was isolated and purified using an RNeasy Mini Kit (Qiagen, Germany) according to the instructions provided by the manufacturer. DNase was used for the removal of any remaining chromosomal DNA. RNA concentrations were measured at 260 nm and 280 nm using a NanoDrop ND-1000 spectrophotometer and stored at –80°C until use. Production of cDNA was carried out using the high-performance cDNA reverse transcription kit from Applied Biosystems (Takara Kit Japan) in 20-µL reaction volumes. Subsequently, 10 µL of 2× SYBR Green PCR Master Mix (Takara Kit Japan) was added to 1 µg of cDNA and 50 nmol (final concentration) of each primer. The expression of genes associated with antimicrobial resistance was quantified using the RT-PCR system (Light Cycler 96 instrument; Roche Diagnostics, USA). The final volume of 20 µL contained 1 µL of cDNA and 1 µL of each primer for the target genes, 10 µL of 2X Q-PCR Master Mix (SYBR, no ROX) (SMOBIO, Taiwan), and 7 µL of distilled water. The *16S rRNA* gene was used as an internal control for the assay. The Δ ΔCt method was used to calculate gene expression. To identify genes with altered expression, the logfold change is calculated on a log 2 scale (equivalent to Δ ΔCt), where log 2 >0 indicates increased expression and log 2 <0 indicates a decrease in expression. Reaction conditions were as follows: initial denaturation at 95°C for 10 min, followed by 40 cycles at 95°C for 30 s and annealing at 58°C for 45 s. Extension was carried out at a temperature of 72°C for 30 seconds.

### Statistical analysis

All analyses were conducted utilizing SPSS version 20 software. The data were subjected to statistical analysis employing the chi-squared test and ANOVA, and differences were deemed significant at a level of p<0.05 using SPSS version 20 (Chicago, IL, USA). 

ANOVA revealed a significant difference between the sub-MIC resistance-suppression concentrations and the negative control for gene expression (p<0.05).

## Results

### Identification of isolates

27 out of 146 isolates were verified to be *S. agalactiae*, accounting for a proportion of 18.5%. These isolates were further classified into 75 specimens originating from Atea Gynaecological Hospital, 53 specimens from Emam Gynaecological Hospital, and 18 specimens from Mehr Central Hospital.

### Antibiotic susceptibility profile

All 27 GBS isolates were individually examined for their susceptibility to each of the antibiotics employed, and it was observed that all the isolates displayed sensitivity towards ceftriaxone, linezolid, and vancomycin, whereas the strains exhibiting the highest percentages of resistance were associated with tetracycline (89%), erythromycin (70.4%), and clindamycin (63%). The strains demonstrated resistance towards chloramphenicol, levofloxacin, penicillin, and ampicillin at rates of 33.3%, 14.8%, 11.1%, and 7.4%, respectively (Table 2 (Tab. 2)).

### Characterization of WO_3_ nanomaterials

**Figure 1:**
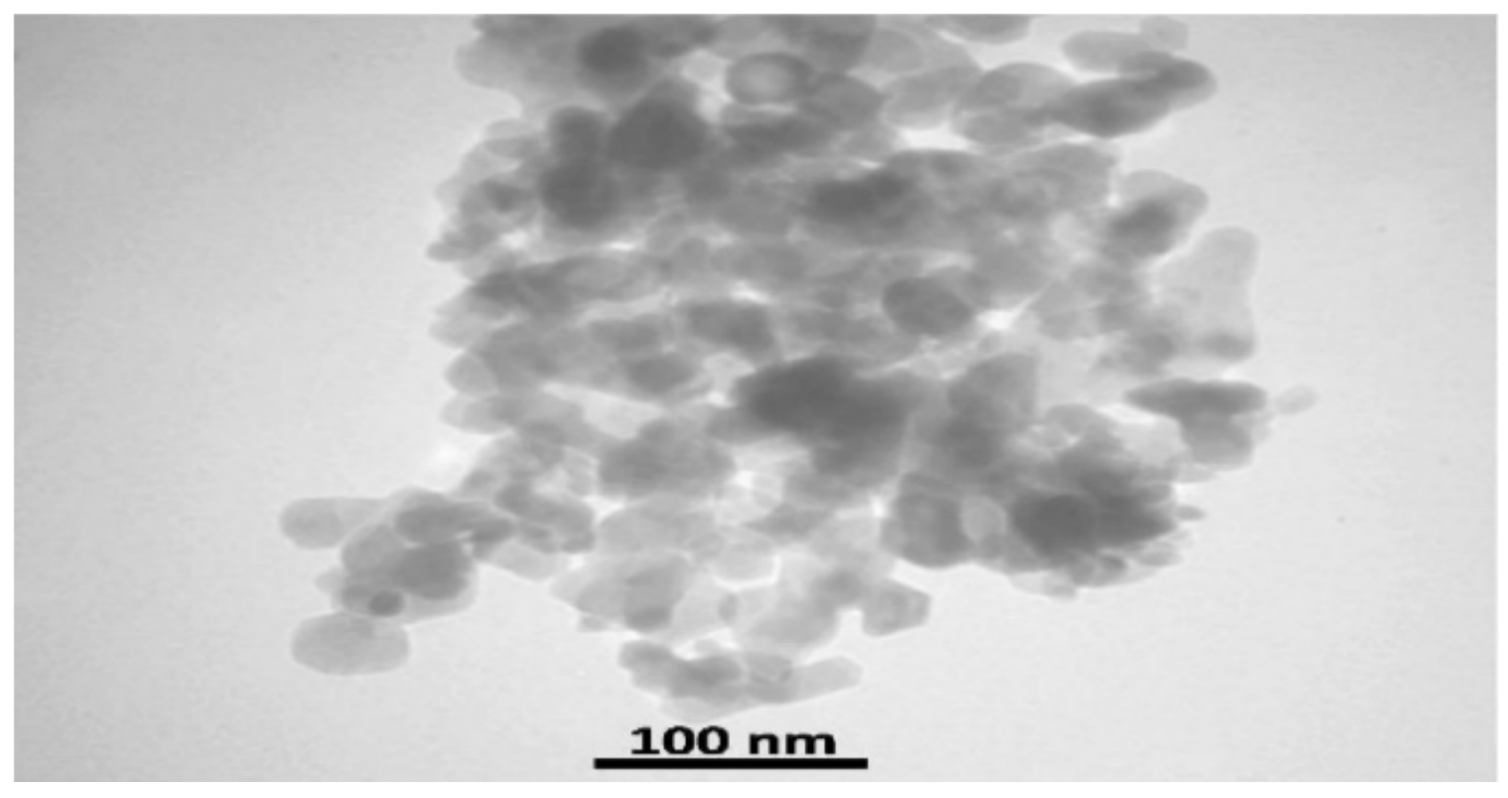
SEM image of WO_3_ at 60,000X magnification.

FE-SEM analysis was performed to assess the particle dimensions and microstructural characteristics. Figure 1 (Fig. 1) depicts the presence of quasi-spherical nanoparticles with an average size of 100 nm in an aqueous sample.

**Figure 2:**
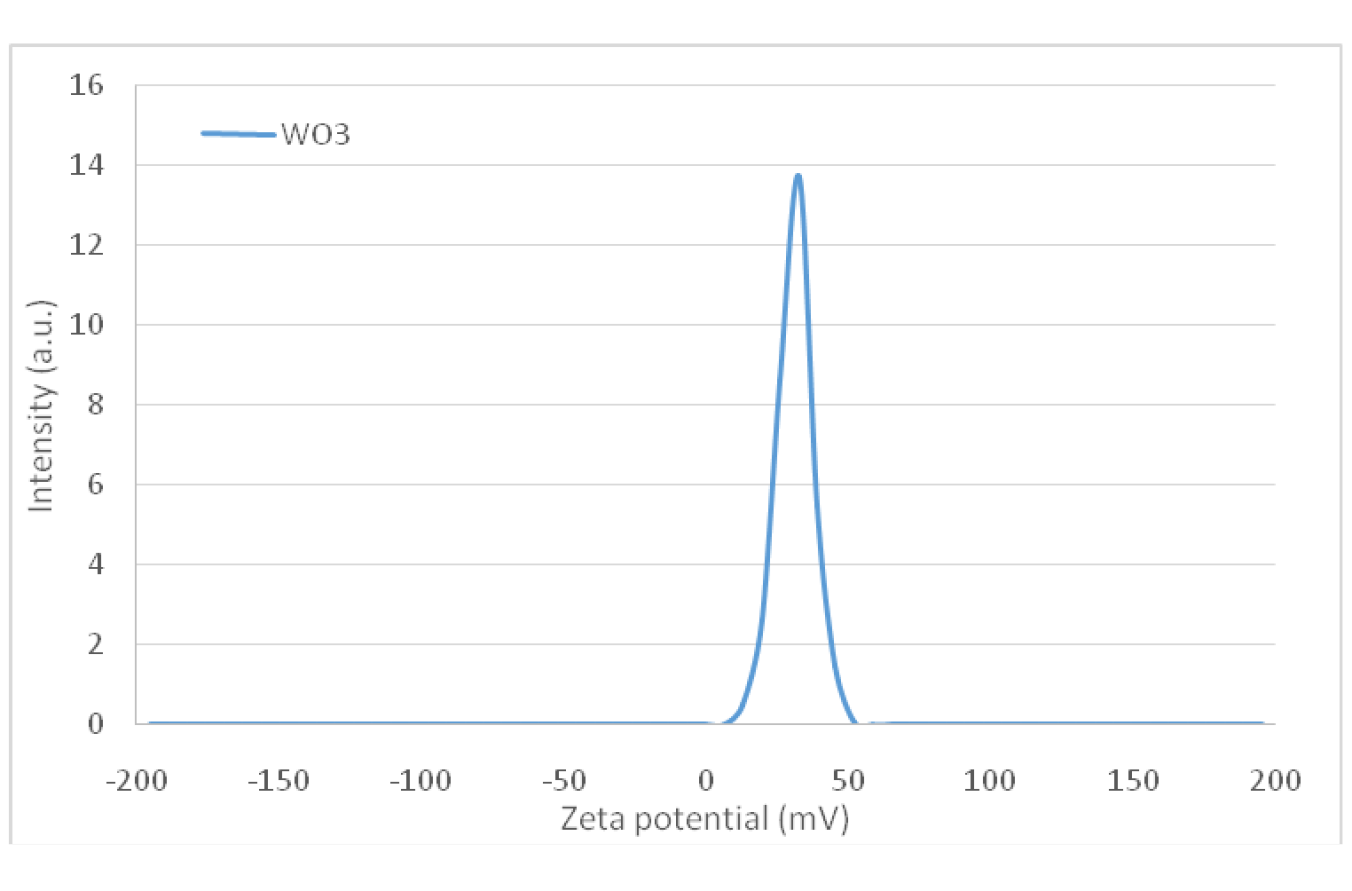
The plot of hydrodynamic diameter of the as-synthesized WO_3_ nanomaterials

**Figure 3:**
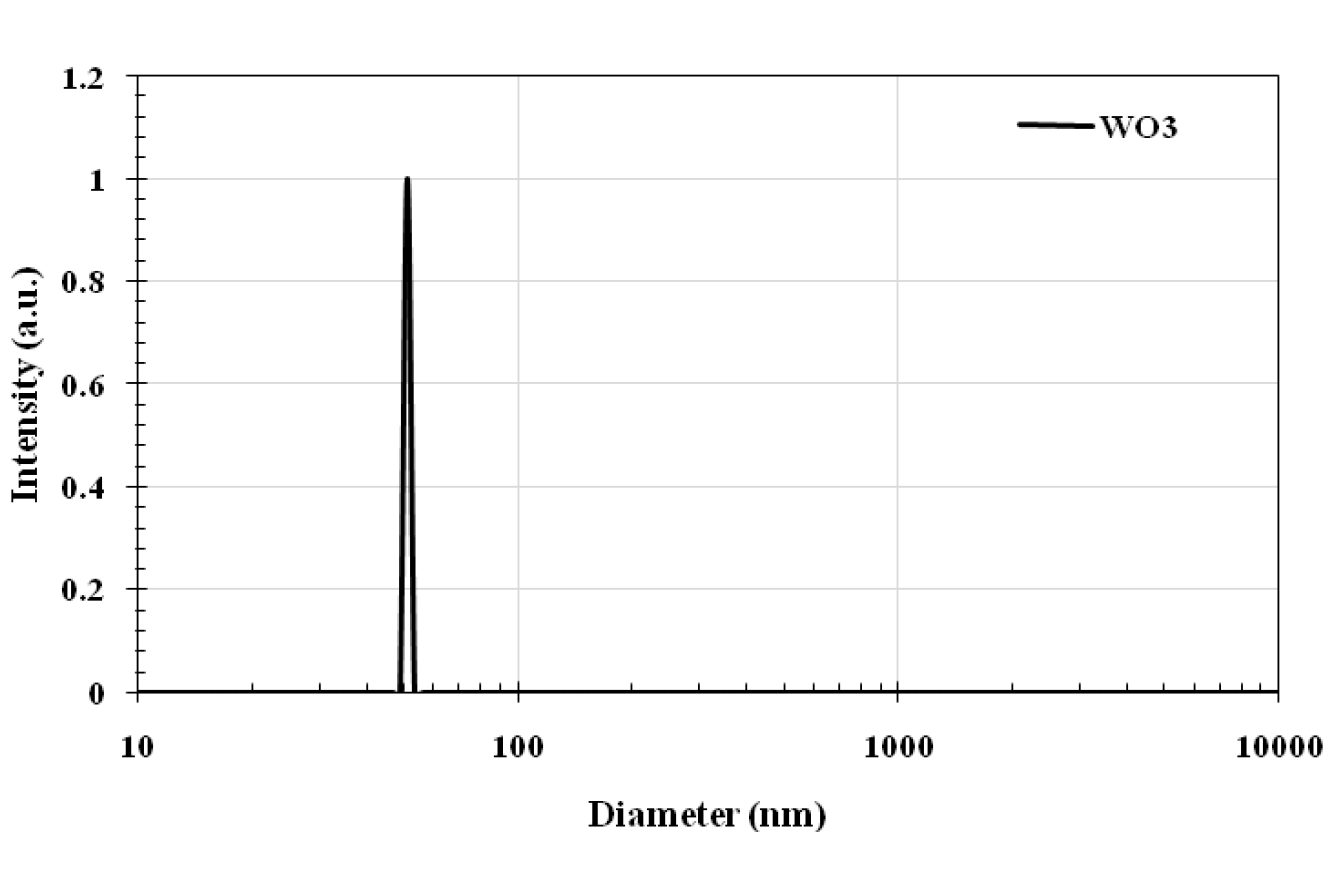
The plot of zeta potential of the WO_3_ nanomaterials

DLS analysis was implemented to determine the stability of the nanostructures. The electric charge present on the surface (known as zeta potential) of particles that are dispersed within a colloidal solution serves as a crucial parameter in assessing the stability of the colloid. The argument commonly put forth is that a reduced absolute magnitude of the zeta potential indicates a diminished stability of colloidal entities, while values surpassing 30 mV signify suspension stability. The absolute zeta-potential of WO_3_ particles was 31.53 mV, meaning that the nanostructure of WO_3_ represents a stable suspension. The hydrodynamic diameter (D_h_) of WO_3_ nanostructures has been calculated to be approximately 51.2 nm. Comparisons reveal that the D_h_ of these nanostructures are larger than those estimated through scanning electron microscopy (SEM) measurements, due to the presence of hydrated layers on their surfaces in an aqueous environment (Figure 2 (Fig. 2) and Figure 3 (Fig. 3)).

### Antibacterial efficiency of WO_3_ nanostructures

After a 24-hour period of incubation under aerobic conditions at 37°C, the test tubes containing WO_3_ nanoparticles at concentrations of 31.25, 62.5, 125, and 250 µg/mL exhibited turbidity, signifying the presence of microbial growth. Conversely, at concentrations of 500, 1,000, 2,000, and 4,000 µg/mL, no turbidity was observed, indicating the suppression of microbial growth. The suspension derived from the tubes with concentrations of 500, 1,000, and 2,000 µg/mL was subsequently inoculated on a BHI agar plate and incubated for 24 hours. 

o growth of bacteria was observed at a concentration of 1,000 µg/mL, thereby confirming the bactericidal nature of the nanoparticles. Moreover, WO_3_ showed variable efficacy against 27 *S. agalactiae* strains with MICs ranging from 500 to 1,000 µg/mL, with 74% having MICs of 500 µg/mL, and 26% a MIC of 1000 µg/mL. To assess the antibacterial effectiveness of WO_3_ particles, a conventional colony count assay was employed. A marginal germicidal effect on *S. agalactiae* strains was observed after 4 hours of treatment with concentrations of WO_3_ particles >500 µg/mL. Following 24 hours of incubation, the survival rates of *S. agalactiae* cells were 84.3% and 68.7% at exposure doses up to 31.25 µg/mL and 62.5 µg/mL, respectively. However, for treatment concentrations up to 250 µg/mL, cell viability diminished to 27.4%, indicating that the ability of WO_3_ nanostructures to eradicate bacteria is concentration-dependent. Our findings demonstrate a statistically significant antibacterial property at higher concentrations of 250–500 µg/mL, as the number of viable cells ranged from 27.4–11%. Furthermore, the antibacterial activity of WO_3_ nanostructures also increased significantly with an increase in exposure time. The survival of *S. agalactiae* cells declined to 43.0%, 34.0%, and 14% after exposure to 31.25, 62.5, and 125 µg/mL WO_3_ nanostructures over a period of 36 h.

### Prevalence of antibiotic resistance genes

The distribution of resistance genes for tetracycline and erythromycin in isolates of *S. agalactiae* was determined using PCR. The findings of our study demonstrated a substantial prevalence of antibiotic resistance in strains of *S. agalactiae* obtained from blood samples, followed by rectovaginal swabs and urine, respectively. In terms of tetracycline resistance genes, the *tetM* gene was the most prevalent, with a frequency of 83.3%, followed by the *tetO* gene at 4.2%. The occurrence macrolide resistance genes was verified in a proportion of the 24 *S. agalactiae* isolates that were classified as resistant/intermediate to erythromycin. Out of these isolates, 9 (37.5%) simultaneously harbored *ermB* and *mefA* genes, 16 (66.6%) were exclusively positive for *ermB*, 1 (4.2%) isolate appeared to be positive for the *mefA* gene alone, and the remaining 8 (33.3%) isolates contained neither *ermB* nor *mefA* genes. The presence of the *tetM* gene was demonstrated in 63.2% (n=12) of the 19 strains that showed resistance towards erythromycin. 

### Effect of WO_3_ nanostructures on the expression of antimicrobial resistance genes

The expression levels of the *tetM* and *ermB* genes were assessed in *S. agalactiae* strains that exhibited resistance to antibiotics and were exposed to sub-MIC concentrations of WO_3_ NPs. The administration of WO_3_ NPs resulted in a significant decrease in the expression of both the *tetM* and *ermB* genes (p<0.05), when compared to the expression of the housekeeping gene (*16S rRNA*), which served as control. This reduction in expression suggests that WO_3_ NPs possess antimicrobial and anti-efflux properties at the transcriptional level. The findings showed that the expression of the *tetM* and *ermB* genes decreased from 2.14±0.34-fold to 0.5±18-fold and from 2.7±0.25-fold to 1.03±0.12-fold, respectively, across a range of 50 to 1.56 µg of tetracycline and erythromycin. ANOVA revealed a significant difference between the sub-MIC resistance-suppression concentrations and the negative control for gene expression (p<0.05).

## Discussion

The presence of Group B streptococci (GBS) in pregnant women is cause for great concern in both public health and clinical settings due to its link to early-onset neonatal disease, stillbirths, mortality, and neurological defects [20]. The present study indicated that the prevalence of GBS colonization among pregnant women was 18.5%. This value was found to be consistent with many previous reports from Jordan, USA, Tanzania, and Brazil, where they ranged between 19.5% and 28.4% [23], [24], [25], [26], but was higher than the reported rates in Iran [27], [28], Ethiopia, Turkey, China, and Korea, where the prevalence ranged from 7.1% to 8.3% [29], [30], [31], [32]. These differences may be attributed to various factors, such as the types of swabs, sample collection methods, composition, microbiological protocols for identifying GBS, and other geographical and timing factors related to screening during pregnancy [20]. Therefore, global variation in maternal GBS colonization rates during pregnancy is likely due to a combination of these factors. Despite the presence of isolates that display resistance to penicillin, ampicillin, and vancomycin, these antibiotics demonstrated efficacy in the management of GBS cases in this study. The percentage of pregnant women with GBS isolates who were susceptible to penicillin and ampicillin (88.8%, 92.6%) in this study is consistent with previous findings [20], [33], [34] in Gondar, Ethiopia (89.8%, 90.8%), Gondar, Ethiopia (88.9%, 90.7%), and Addis Ababa, Ethiopia (85.5%, 85.4%). Furthermore, the percentage of GBS isolates susceptible to vancomycin obtained in this study (100%) is higher than that in Gondar (83.7%), Gondar (83%), and Addis Ababa (96.3%) [20], [33], [34]. This finding aligns with the results from previous studies conducted by Engelbrecht [35] and Haimbodi et al. [36], where susceptibility rates of 100% were reported for ceftriaxone, vancomycin, and linezolid. However, the CLSI standards [19] as well as related studies elsewhere reported the absence of GBS isolates resistant to penicillin, ampicillin, and vancomycin [37], [38], [39]. This consistency in findings across different studies highlights the effectiveness of these antibiotics in treating GBS infections. However, it is important to note that susceptibility patterns may vary geographically and over time, so regular monitoring and surveillance are crucial to ensure appropriate antibiotic selection for GBS infections. Clindamycin and erythromycin have been proposed as substitute antibiotics for pregnant women allergic to penicillin who face a heightened risk of anaphylaxis [40]. Nevertheless, recent studies have sparked worldwide apprehension regarding the escalating antimicrobial resistance witnessed in GBS strains towards these particular antibiotics [40]. Our analysis identified a resistance frequency of 70.3% for erythromycin and 63% for clindamycin. It is important to note that the prevalence of resistance to erythromycin among GBS strains observed in our research was higher than in similar investigations also conducted in Iran [41], [35], [36]. Nevertheless, notably higher levels of erythromycin resistance in GBS have been documented in nations such as China (92.5% and 84.6%), Iraq (58.6%), and the USA (50.7%) [42], [43], [44], [45]. Furthermore, increased levels of clindamycin resistance in GBS strains have been reported in Iran, China, Iraq, and Italy [31], [42], [44], [46]. These outcomes underscore the importance of conducting susceptibility assessments on GBS isolates before commencing prophylactic treatment with erythromycin or clindamycin, in accordance with the recommendations put forth by the CDC. The GBS isolates obtained from the pregnant women in this study demonstrated non-susceptibility rates of 89% to tetracycline. These high rates of non-susceptibility align with the overall estimate of tetracycline resistance in Africa (82.6%) as well as findings from previous studies in Gondar (73.4%), Ethiopia in general? (79.6%), and Addis Ababa (90.2%) [33], [34], [47]. Similarly, a systematic review conducted by Hayes et al. [8] reported resistance rates to tetracycline of >80%. The increasing prevalence of GBS isolates with non-susceptibility to tetracycline, as observed in this study and supported by previous research, raises concerns about the continued use of tetracycline for both treatment and prophylaxis. The substantial resistance of GBS to tetracycline can be linked to the remarkable frequency of the *tetM* gene (83.3%), as shown in the present results. The increased prevalence of the *tetM* gene can be ascribed to the extensive distribution of *tet* genes within pathogens, opportunistic pathogens, and constituents of the indigenous microbiota [9]. The rise in tetracycline resistance is further linked to the cost-effectiveness of the antibiotic, which is extensively utilized for prophylactic purposes in the management of animal and human infections [48]. The *tetO* gene is characterized by a relatively low prevalence of 4.2%, indicating its rarity within tetracycline-resistant GBS isolates. Similar findings were reported in Kuwait, where some authors observed a percentage of 89.5% tetracycline-resistant isolates. These isolates carried the *tetM* gene in approximately 94.5% of cases and the *tetO* gene in around 3.9% of cases [49]. However, Rojo-Bezares et al. [50] demonstrated that 67.6% of their isolates carried *tetM*, while 25% of them also carried *tetO*, and 5.9% were found to have both *tetM* and *tetO* genes [51]. The corresponding rates in China [52] were 92%, 5% and 1%, whereas 88.8% of isolates recovered from Namibia proved to be *tetM*-positive and none of them possessed the *tetO* gene [39]. PCR analysis revealed that out of 24 GBS samples exhibiting intermediate and resistant characteristics towards erythromycin, 66.6% (16/24) tested positive for the *ermB* gene and 4.2% (1/24) for the *mefA* gene. The prevalence of the *ermB* determinant indicates that GBS frequently employ target methylation as the mechanism for developing resistance against macrolide antibiotics. Multiple studies conducted in different countries, including Italy [46], South Africa [7], USA [53], Iran [54], and France [55], have reported similar results. In these studies, it was observed that the *ermB* gene is most widely distributed among GBS strains. Moreover, the current research corroborates the findings reported by Poyart et al. [24], concerning the rarity of the *mefA* gene in GBS isolates, suggesting that efflux pumps facilitated by this gene are not a predominant mechanism in the development of resistance to macrolide antibiotics. 

The literature seldom discusses the antimicrobial properties of tungsten oxide, in spite of its utilization in water purification. Previous studies have suggested that tungsten oxide displays photocatalytic properties under visible light exposure [56]. In the case of tungsten oxide, the reduction of pathogenic microorganisms can be attributed to a process known as photokilling. Photokilling is a process by which reactants damage cell membranes, releasing intracellular compounds. These compounds are then subject to oxidation by photo-catalysis [50], [57], [58]. In the context of photocatalysts, electron-hole pairs are generated when energy is applied across the band gap. They react with oxygen and water to form superoxide anion radicals (O_2_●–) and hydroxyl radicals (●OH). These oxidative species are thought to be the main cause of bacterial cell death, as they are extremely reactive [59]. The findings from the study by Matharu et al. [60] ireveal that the efficacy of tungsten oxide nanoparticles in deactivating *S. aureus* surpasses its effectiveness compared to *E. coli* when exposed to visible light. Those authors inferred that the variation in antimicrobial effectiveness may be due to differences in cell wall structure. Additionally, the differences in potency may be associated with the varying affinities of microbial cell walls for tungsten oxide. As a result, a greater number of hydroxyl radical assaults is required to achieve complete bacterial inactivation in Gram-negative bacteria. 

Metallic nanoparticles offer a novel approach in addressing antimicrobial resistance (AMR) owing to their distinct mechanisms. These mechanisms include the disruption of bacterial cell membrane potency and integrity, the inhibition of biofilm development, the generation of reactive oxygen species (ROS), the augmentation of the host immune responses, and the suppression of RNA and protein synthesis by stimulating intracellular processes [61]. In this regard, there are a limited number of studies on the antimicrobial activity of tungsten oxide (WO_3_) nanoparticles. Some researchers highlight the potential of WO_3_ nanoparticles as a promising antimicrobial agent. Duan et al. [18] have shown impressive effectiveness against both *Escherichia (E.) coli* and *Staphylococcus (S.) aureus*. SEM and transmission electron microscopy (TEM) images have confirmed the damage to bacterial cell membranes, as evidenced by molecular dynamics simulations. The germicidal activity of WO_3_-x nanodots was further enhanced when exposed to simulated sunlight. Combining a 30-minute exposure to sunlight with 50 µg/mL WO_3_-x nanodots resulted in a 70% reduction in *E. coli* viability. Electron spin resonance (ESR) spectroscopy disclosed the production of hydroxyl radical species, which serve as the fundamental mechanism behind this photocatalytic activity. Additionally, the biocompatibility of WO_3_-x nanodots on eukaryotic cells has been validated. In short, these findings strongly suggest that WO_3_-x nanodots hold significant potential in antibacterial applications while maintaining a broad level of biocompatibility [14]. The study conducted by Jeevitha et al. [62] discovered that the tungsten oxide-graphene oxide (WO_3_-GO) nanocomposite exhibits antibacterial activity, with minimum inhibitory concentration (MIC) values ranging between 2.5 and 5 mg/mL. Analysis using field emission scanning electron microscopy (FE-SEM) demonstrated notable morphological alterations in the bacterial membranes of *E. coli *and* Bacillus subtilis* when exposed to the irradiated samples containing the WO_3_-GO nanocomposite. The present study represents a pioneering investigation into the impact of tungsten oxide nanoparticles on gene expression associated with antibiotic resistance. The nanostructure treatment induced an important reduction in the expression of *ermB*, and *tetM* resistance genes, indicating its potential as an effective strategy to combat antibiotic resistance. Within the scope of the influence of metal nanoparticles on gene expression, Dolatabadi et al., unveiled the inherent capability of AgNPs produced through biosynthesis in combatting ciprofloxacin-resistant *K. pneumoniae* strains. These nanoparticles impede the proliferation of these strains and also reduce the expression of the *OxqAB* genes, which compose the multidrug efflux pump. The MIC of artificial AgNP significantly affects the level of expression of the *OxqA* gene, indicating that biosynthesized AgNPs could disturb the operation of the multidrug efflux pump, rendering the bacteria more vulnerable to antibiotic treatment [63]. In addition to the previously mentioned finding, Nejabatdoust et al. [64] discovered that combining ZnO NPs and ciprofloxacin (CIP) significantly increased the strains susceptibility to various antibiotics, such as methicillin, erythromycin, and tetracycline. This implies that the combination of ZnO NPs and CIP has the potential to address drug resistance in *S. aureus* by reducing the expression of efflux pump genes (*norA, norB, norC, tet38*) and enhancing antibiotic susceptibility. In a notable contribution to the field, Nejabatdoust et al. [64] provided invaluable insights into the development of innovative strategies to combat antibiotic resistance in *S. aureus* infections, with a particular focus on the role of efflux pump genes. Lotfpour et al. [65] demonstrated that iron nanoparticles inhibited the expression of the* TEM-1* β-lactamase gene and impeded the proliferation of *P. aeruginosa*. The results demonstrated that the expression levels of the *TEM-1* gene in two isolates subjected to Iron oxide nanoparticles (IONPs) exhibited a reduction of 78% and 75%, respectively, in comparison to the levels observed in the untreated bacteria [64]. Moreover, the *norA* gene was found to be expressed at a markedly diminished level among clinical and standard strains treated with an Ag-doped CuFe2O4 nanocomposite in conjunction with ciprofloxacin, exhibiting a reduction of 59% and 65%, respectively. It can be concluded that the decrease in the *norA* gene among the bacteria treated with ciprofloxacin and nanocomposites can be attributed to two factors. Firstly, the inhibition of bacterial transcription caused by the antibiotic is a contributory factor. Secondly, the inhibition of bacterial enzymes via direct interaction with the nanocomposite represents an additional contributory factor. It is worth noting that the decrease in efflux pump gene activity may be linked to the inhibition of bacterial gene transcription by reactive oxygen species (ROS) and/or the direct interaction between the nanoparticles (NPs) and the transcription factors, which can interfere with their ability to bind to DNA and initiate gene transcription [66].

## Conclusion

The prevalence of *S. agalactiae* colonization among pregnant women in this study is relatively high, and this increases the risk of serious neonatal infections. These findings support the need for pregnant mothers to undergo screening for this organism. Additionally, it is crucial to administer appropriate antibiotic prophylaxis during pregnancy in preventing early-onset *S. agalactiae* neonatal infections and associated complications. The emergence of antibiotic resistance to erythromycin and clindamycin in GBS isolates is a growing concern; hence, susceptibility testing of isolates before initiating prophylaxis with these antibiotics is necessary. Further studies are needed to determine the correlation between different risk factors and maternal GBS colonization during pregnancy in different geographic regions. The potential of WO_3_ nanoparticles as a viable solution for addressing antibiotic resistance in *S. agalactiae* infections is shown by the results. These findings open new possibilities for the development of innovative antibacterial agents. By harnessing the unique properties of WO_3_ nanoparticles, researchers can explore novel approaches to combatting antibiotic resistance in this specific type of infection. This avenue of research holds promise for the future, as it offers a potential alternative to traditional antibiotics and could contribute to the fight against antibiotic-resistant bacteria. Further studies and experiments are needed to fully understand the mechanisms by which WO_3_ nanoparticles exert their antibacterial effects and to explore their potential applications in clinical settings.

## Notes

### Competing interests

The authors declare that they have no competing interests.

## Figures and Tables

**Table 1:**
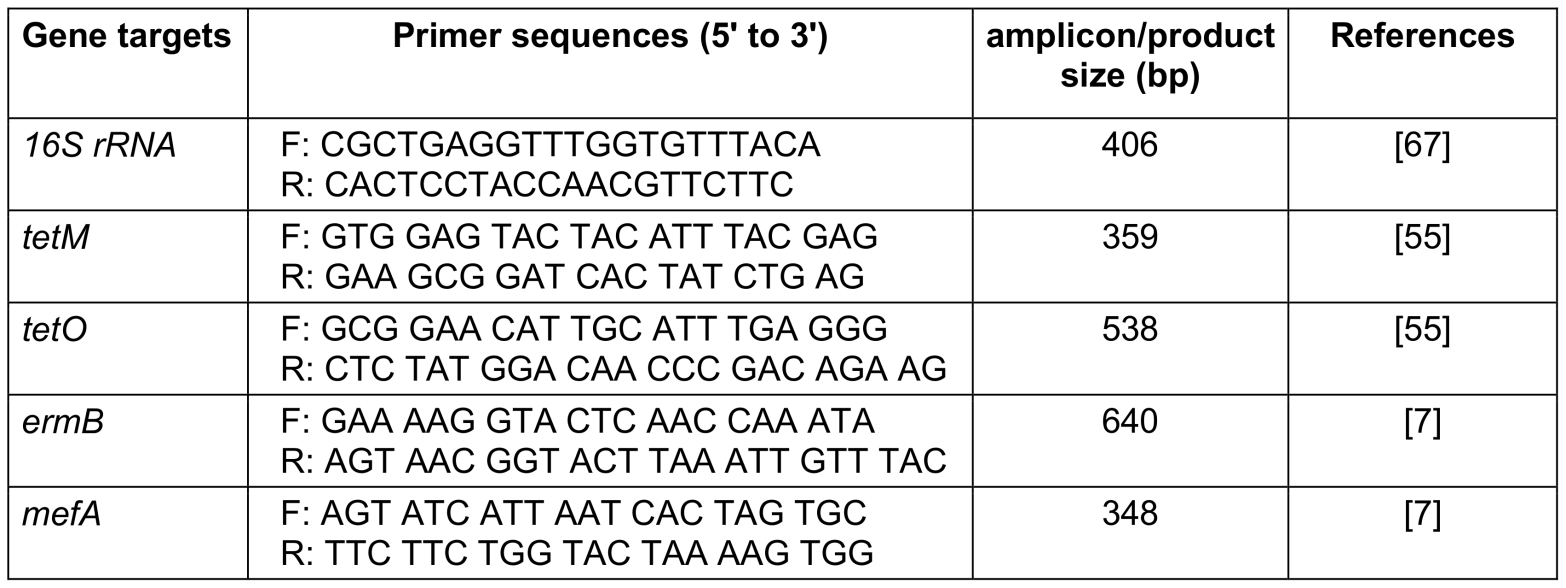
Primers used in the current study

**Table 2:**
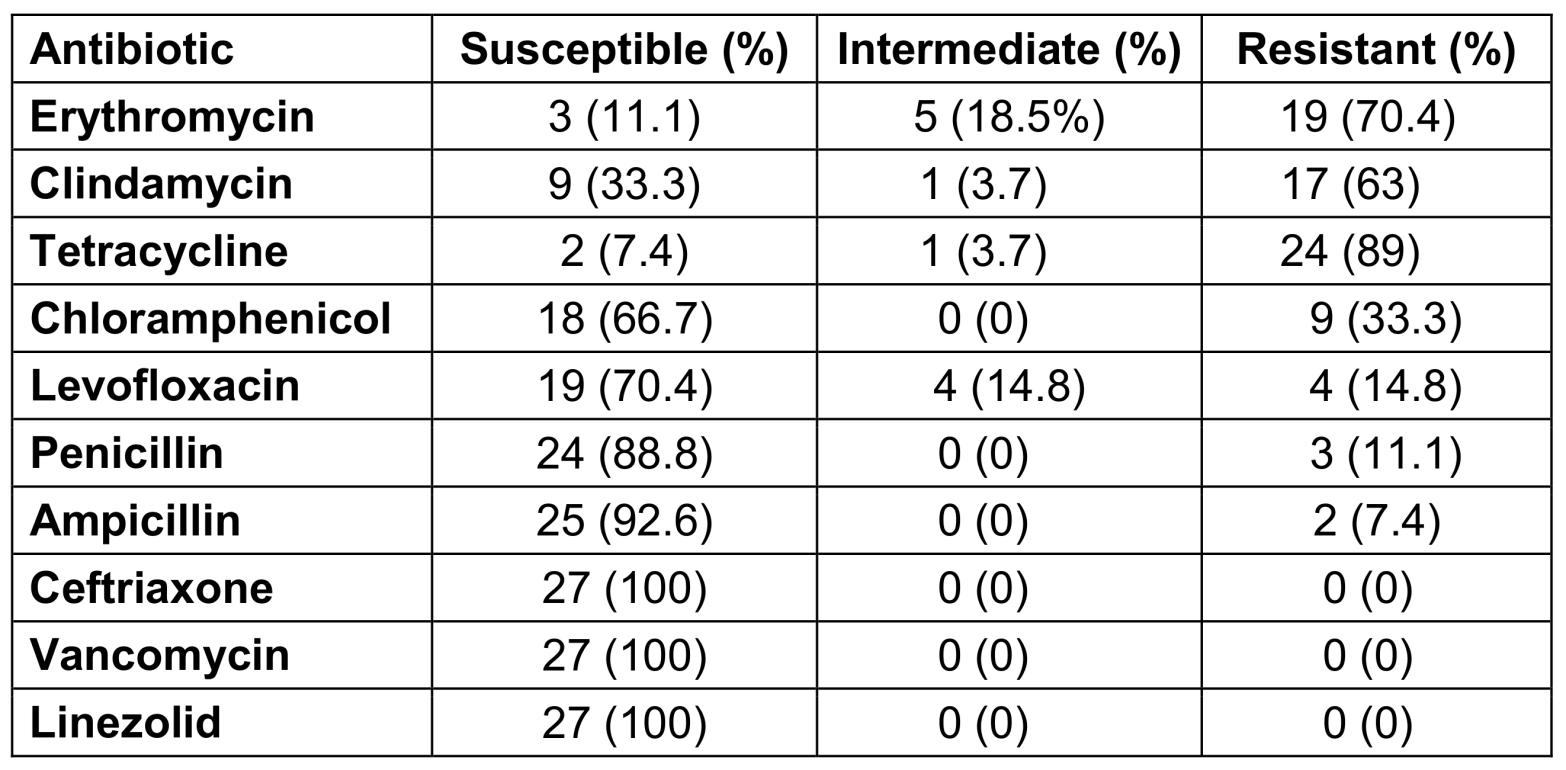
Antimicrobial susceptibility patterns of GBS isolates
